# Liquid Level Detection in Standard Capacity Measures with Machine Vision

**DOI:** 10.3390/s21082676

**Published:** 2021-04-10

**Authors:** Gregor Bobovnik, Tim Mušič, Jože Kutin

**Affiliations:** Laboratory of Measurements in Process Engineering, Faculty of Mechanical Engineering, University of Ljubljana, Aškerčeva 6, SI-1000 Ljubljana, Slovenia; maticko.music@gmail.com (T.M.); joze.kutin@fs.uni-lj.si (J.K.)

**Keywords:** liquid level, standard capacity measure, legal metrology, machine vision, parallax

## Abstract

Capacity measures are commonly used volume standards for testing measuring systems for liquids other than water. Manual readings from the measuring scale can often be difficult due to the location of the capacity measure or to the nature of the measured liquid. This article focuses on the automation of this procedure by using a single camera machine vision system. A camera positioned perpendicular to the transparent neck captures the image of the liquid meniscus and the measuring scale. The volume reading is determined with the user-defined software in the LabVIEW programming environment, which carries out the image preprocessing, detection of the scale marks and the liquid level, correction of lens distortion and parallax effects and final unit conversions. The realized measuring system for liquid level detection in standard capacity measures is tested and validated by comparing the automated measurement results with those taken by the operators. The results confirm the appropriateness of the presented measuring system for the field of legal metrology.

## 1. Introduction

Capacity measures are commonly used volume standards for the testing and inspection of measuring systems for liquids other than water. Depending on their nominal volume, the capacity measures can be categorized into the following [1]: standard glass flasks (≤10 L), standard test measures (≤20 L) and prover tanks (up to several thousand liters). Despite the fact that the last two types are made of steel, their neck part is made of a glass tube, or has glass plates or separate and fixed gauge glasses [1]. They are all equipped with scale marks corresponding to their nominal capacity and to at least 1% below and above the nominal capacity [1]. For type approval tests, the requirement for the expanded uncertainty of the determination of errors on the indication of the measured quantity is one-fifth of the maximum permissible error, i.e., 0.1% for measuring systems with an accuracy class of 0.5 [2]. For all capacity measures, the diameter of the neck or of the glass gauge should be large enough to minimize the capillary and meniscus effects; however, the reading of the meniscus (its curvature and fluctuations) represents an important contribution to the uncertainty and repeatability of the volume determined by standard capacity measures [3,4]. The operator needs to be certain that the reading uncertainty is small enough in view of the requirement for the combined expanded uncertainty.

Manual (visual) liquid level detection and obtaining readings from the measuring scale can often be time consuming and difficult due to the location of the capacity measure or the nature of the measured liquid. As the capacity measures are calibrated against their scale marks, any automated measuring system also must detect the liquid level with respect to the attached measuring scale. Therefore, it is evident that the automation of such measuring procedures cannot be realized using the traditional techniques for liquid level measurement (such as capacitance or pressure transducers, ultrasonic and radar methods, tuning forks [5]). An alternative utilizes the application of machine vision, where a camera is used for image acquisition, and computer vision techniques are used for processing the acquired images. The aim of this article is to present a simple, configurable and low-cost machine vision system capable of reading the liquid level with respect to the attached measuring scale, which is applicable for the purposes of legal metrology. It consists of a standard web camera and a PC including the LabVIEW software for image processing. The developed system compensates for the lens distortion and parallax effect [6,7], which are consequence of lens imperfections, physical properties of light and some distance between the measuring scale of the capacity measure and the liquid column. The magnitudes of these corrections are dependent on geometrical distances in the measuring system, which means that the machine vision system must be configured and validated for each individual application. Due to this, the presented measuring systems are especially employable for liquid level measurement in fixed applications, such as tank provers.

In the available literature, we can find some similarly configured machine vision measuring systems with a single camera that are used in other applications requiring the detection of liquid level. Chakravarthy et al. [8] and Gaber et al. [9] employed a light source that illuminated the surface of the liquid, while the corresponding light pattern on the surface of the liquid was imaged by a digital camera. In a similar system, the liquid level detection in a tank is based on the pixel count of the float in the captured image [10]. Another type of measuring system for water levels in rivers uses the image of the level gauge immersed in water, which is captured by a laterally positioned camera [11,12,13]. Refs. [14,15,16,17] present machine vision systems for the detection of liquid levels in applications similar to the one we are interested in. Pithadiya et al. [14] present an application for the detection of under- or overfilled bottles with emphasis on edge detection algorithms, while Eppel and Kachman [15] inspect the applicability of computer vision techniques for the recognition of liquid levels in various transparent containers. A later study suggests that good accuracy of liquid–air boundaries can be achieved with various edge detection methods. Samman [16] presents a LabVIEW based system for liquid level and colour monitoring in glass cylinders during chemical reaction. Liu et al. [17] expose the importance of lighting conditions in machine vision systems used for the detection of a water–oil interface.

This paper is structured as follows. Section 2 presents the background of the parallax correction model, including the effects of a tilted camera. Section 3 describes the test setup and the signal processing algorithm, which also includes image correction for lens distortion and parallax effects. In Section 4, the measuring system for liquid level detection in standard capacity measures is experimentally tested and validated by comparing the automated measurement results with those taken by two operators.

## 2. Parallax Correction

Due to the distance between the measuring scale of the capacity measure and the liquid column, every optical system is generally susceptible to parallax effects [5]. The parallax is a shift of the apparent position of the liquid level in the background, regarding the measuring scale positioned in the foreground. When the liquid level is not aligned with the central axis of the camera, the parallax error occurs. This effect can be reduced by adjusting the camera so that it remains aligned with the liquid level, or by considering corrections after the image is captured. In this article, we use the camera in a fixed position and perform corrections of the parallax during image processing, which is crucial for achieving the accuracy of the measured values. This section presents the theoretical foundations for the parallax correction of two different cases: (i) parallax correction for the camera with its centreline positioned perpendicular to the measuring scale and (ii) parallax correction for the tilted camera.

### 2.1. Parallax Correction for the Camera Centreline Perpendicular to the Measuring Scale

**Figure 1:**
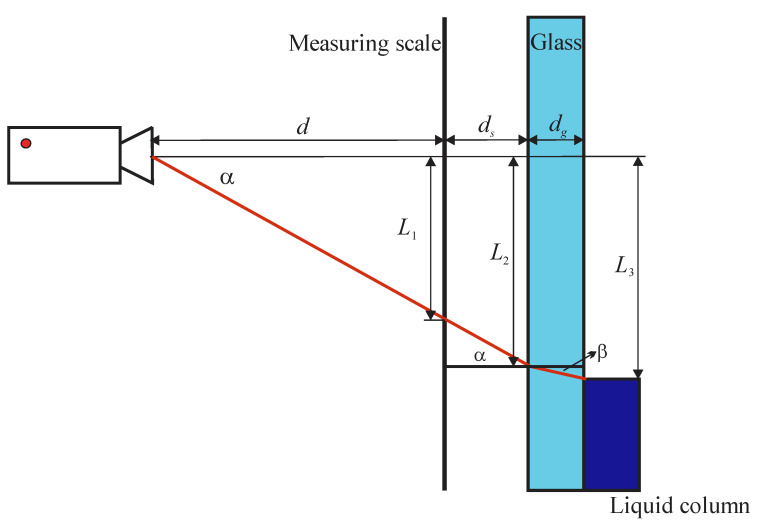
Scheme of the parallax effect.

Assuming that the camera is positioned perpendicular, as shown in Figure 1, the actual liquid level *L*_3_ is not equal to the apparent position of the liquid level *L*_1_ read by the camera from the measuring scale. The determination of the actual liquid level *L*_3_ is affected by the distance between the camera and the measuring scale (*d*), the distance between the measuring scale and the glass neck (*d_s_*), and by the glass thickness (*d_g_*).

According to Figure 1, the liquid level *L*_3_ can be calculated as follows:

(1) $$L_{3}=L_{2}+d_{g}\tan\beta$$

where *L*_2_ can be related to *L*_1_ by a similar triangle principle:

(2) $$L_{2}=L_{1}\frac{d+d_{s}}{d}$$

and the angle β is defined according to Snell’s law by the ratio between refractive indices of air (*n*_1_ ≈ 1) and glass (*n*_2_):

(3) $$\sin\beta =\frac{n_{1}}{n_{2}}\sin\alpha$$

The refraction index of glass is higher than that of air (*n*_2_ > *n*_1_); therefore, the angle of refraction β is smaller than the angle of incidence α. Expressing the angle α as $\alpha =\arctan\left(L_{1}/d\right)$, the liquid level *L*_3_ can be expressed as:

(4) $$L_{3}=L_{1}\left(1+\frac{d_{s}}{d}\right)+d_{g}\tan\left(\arcsin\left(\frac{n_{1}}{n_{2}}\sin\left(\arctan\left(\frac{L_{1}}{d}\right)\right)\right)\right)$$

**Figure 2:**
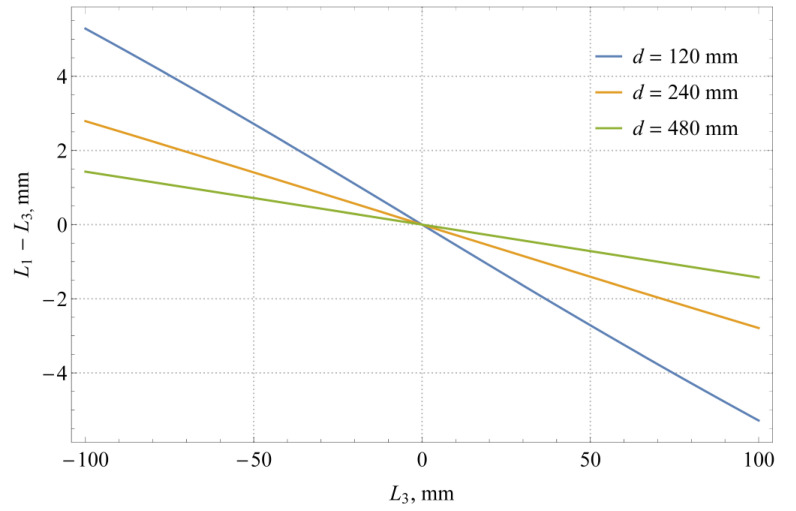
Magnitude of the parallax effect *L*_1_ − *L*_3_ (simulation made for *d_s_* = 5 mm, *d_g_* = 3 mm, and *n*_2_/*n*_1_ = 1.52).

Observe how certain terms in Equation (4) influence the measurement result. Figure 2 shows the influence of the camera distance *d* on the magnitude of the parallax effect, defined as the difference between the scale reading *L*_1_ and the actual liquid level *L*_3_. The parallax is more pronounced for smaller distances between the camera and the measuring scale.

**Figure 3:**
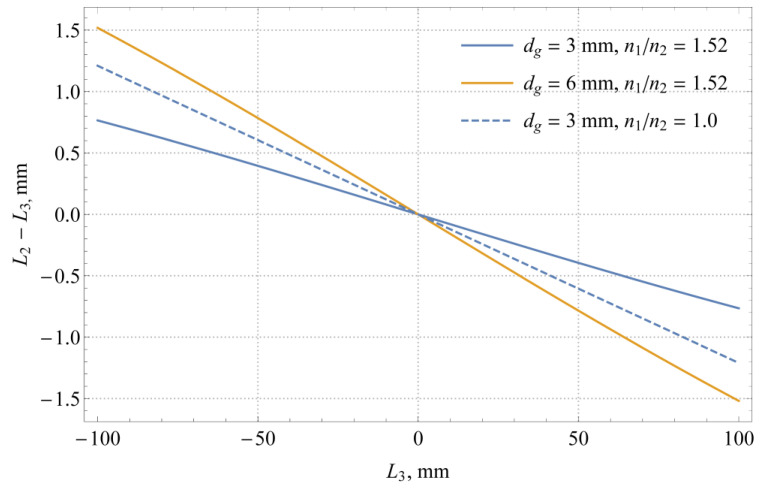
Magnitude of parallax effect *L*_2_ − *L*_3_ (simulation made for *d =* 240 mm and *d_s_* = 5 mm).

Figure 3 demonstrates a contribution from the glass wall (neck) to the parallax correction, which results in a difference between the level *L*_2_ and the liquid level *L*_3_. This contribution includes the effects of finite glass thickness and the refractive index of glass. As expected, the parallax effect increases with glass thickness. The light refraction in the glass results in a smaller magnitude of the parallax effect; therefore, if we were to neglect the refractive index of glass, i.e., *n*_2_ = *n*_1_, this would mean overcorrecting the camera readings. According to [3], the flasks and glass gauges are usually made of borosilicate (*n*_2_ ≈ 1.48) or soda-lime glass (*n*_2_ ≈ 1.52). By comparing the solid and dashed blue curves in Figure 3, it can be seen that if the distance between the measuring scale and liquid remains the same, the glass itself reduces the parallax by approximately 0.4% in the considered case. For the same case, the overall parallax effect amounts to ±0.8 and ±1.5% for glass thicknesses of 3 and 6 mm, respectively.

### 2.2. Parallax Correction with Tilted Camera

**Figure 4:**
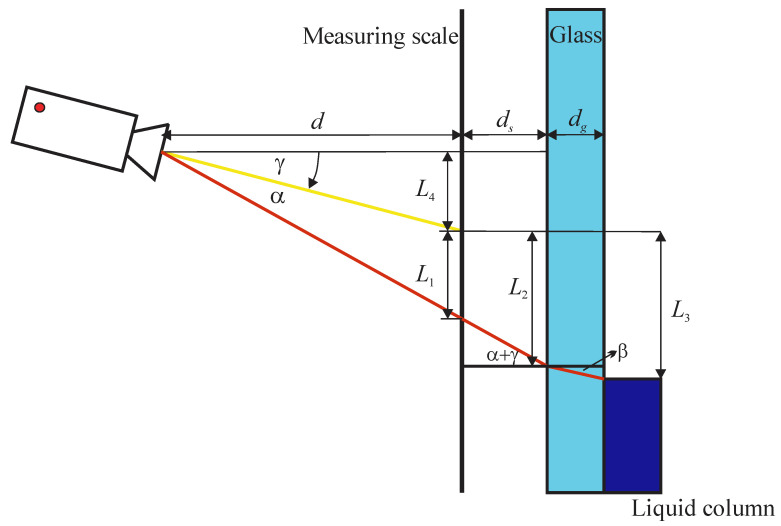
Scheme of the parallax effect for the tilted camera.

If the centreline of the camera and the measuring scale are not perpendicular, the parallax correction model from Section 2.1 needs to be upgraded with the effect of the camera tilt angle. The effect of the tilt angle γ, which is measured in a clockwise direction, is schematically presented in Figure 4.

According to Figure 4 and using Equation (4), the liquid level *L*_3_ is equal to the following:

(5) $$L_{3}=L_{1}+d_{s}\tan\left(\alpha +\gamma \right)+d_{g}\tan\left(\arcsin\left(\frac{n_{1}}{n_{2}}\sin\left(\alpha +\gamma \right)\right)\right).$$

Since the distance *L*_1_ is measured from the centreline of the camera, which is no longer horizontal, we must calculate the viewing angle (α + γ). This can be determined as:

(6) $$\tan\left(\alpha +\gamma \right)=\frac{L_{1}+L_{4}}{d},$$

where *L*_4_ is equal to the following:

(7) $$L_{4}=d\tan\gamma .$$

Inserting Equations (6) and (7) into Equation (5), liquid level *L*_3_ can be expressed as:

(8) $$L_{3}=L_{1}\left(1+\frac{d_{s}}{d}\right)+d_{s}\tan\gamma +d_{g}\tan\left(\arcsin\left(\frac{n_{1}}{n_{2}}\sin\left(\arctan\left(\frac{L_{1}}{d}+\tan\gamma \right)\right)\right)\right).$$

**Figure 5:**
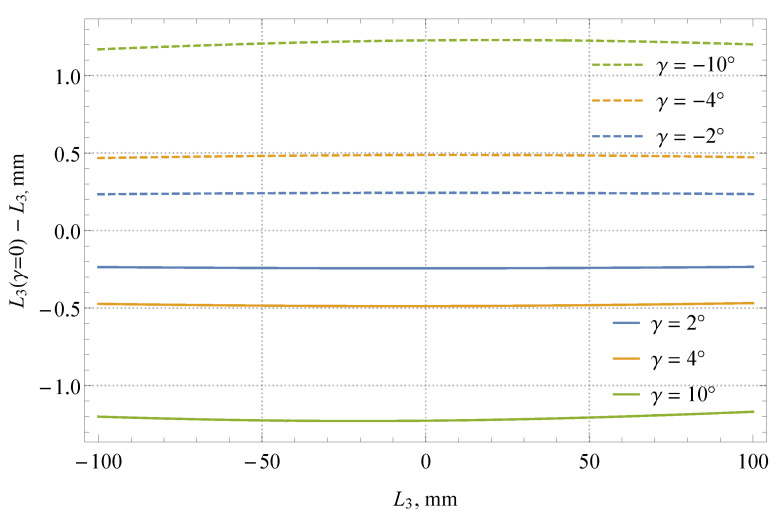
Difference of parallax effects for a camera positioned perpendicular to the measuring scale and for a tilted camera (simulation made for *d* = 240 mm, *d_s_* = 5 mm, *d_g_* = 3 mm, and *n*_2_/*n*_1_ = 1.52).

Figure 5 shows the difference between the parallax effect for γ = 0 and the parallax effect for three different tilt angles of the camera in both directions. The observed difference increases with the tilt angle; it is negative for positive tilt angles and positive for negative tilt angles. One can see that the camera tilt effect has small nonlinearity over the range of simulated liquid levels. Additional simulations showed that this nonlinearity becomes more significant with the decreasing distance *d*. The presented results can be interpreted as a contribution of the tilt angle uncertainty to the uncertainty of the measured liquid level (related to the inability to position the camera ideally perpendicular to the measuring scale).

## 3. Measuring System

The machine vision system was tested in a laboratory environment. Although the developed measuring system is mainly intended for measurements of liquids other than water, water was chosen as a testing medium to conduct the level detection of more transparent liquids. The measurements were performed on the standard capacity measure with a nominal capacity of 100 L (Aleksander Lozar). The measuring scale division represents 10 mL, which is equal to 0.01% of the nominal capacity, and one scale division is approximately 1 mm. Images were captured using a standard web camera (Razer, Kiyo, resolution: 1920 × 1080 pixels) and processed in LabVIEW Vision Development Module. The camera was fixed and positioned perpendicular to the measuring scale (γ ≈ 0°) with its centre close to the zero-scale mark. The perpendicular position was achieved by ensuring that the vertical distances measured in pixels in the acquired image between the scale mark *i*(0) in the centre of the image and the scale marks, which are at some given distance ±∆*i* from *i*(0), were equal.

**Figure 6:**
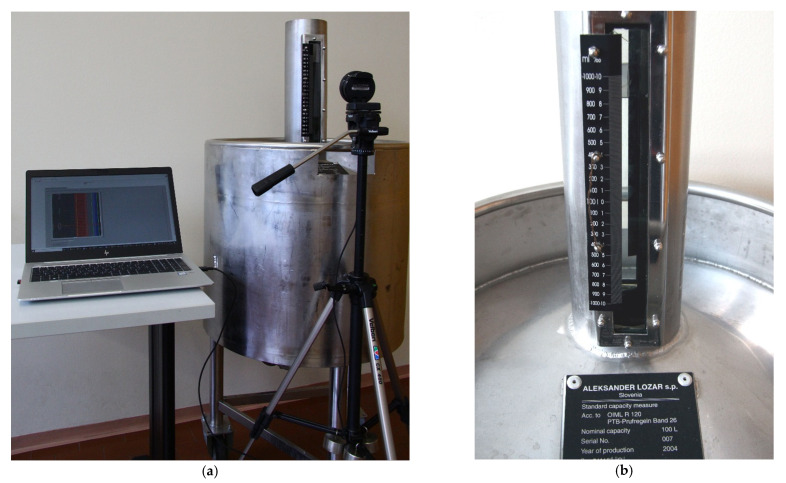
Experimental setup of the measuring system (**a**) and the measuring scale of the capacity measure (**b**).

Figure 6 shows the measuring system with the camera and the capacity measure under tests. All parameters of the measuring system are defined in Table 1. The distances were measured using a measuring tape and a slide gauge. The average distance between the scale marks was determined by dividing the length of the measuring scale by the total number of scale divisions.

**Figure 7:**
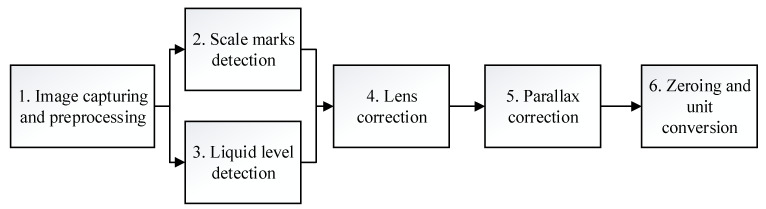
Operational diagram of the computer program.

Figure 7 shows the operational diagram of the computer program used to determine the liquid level from the captured images, which comprises the following steps.

Step 1: Image Capturing and Preprocessing

The process of liquid level determination is initiated by acquiring the image of the liquid column in the capacity measure with the transparent neck. Once the image is obtained, it is converted into a monochrome image, which is helpful for further processing (edge detection). In the case of transparent liquid, it does not matter which (monochrome) colour plane is used, but with coloured liquids, choosing the right colour plane could provide more reliable and consistent results.

Step 2: Scale Mark Detection

**Figure 8:**
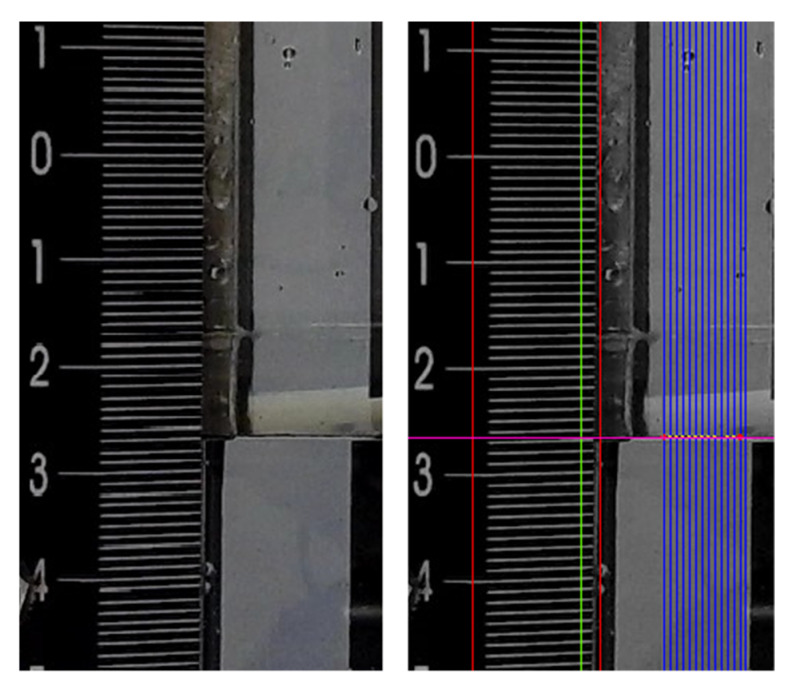
Measuring scale and liquid level; raw image (left) and processed image with marked detection area/lines (right).

The reading of the marks of the measuring scale in the acquired image is carried out in several steps. First, the measuring scale is detected either by using pattern recognition or within the predefined region of interest, if the camera is expected to remain in a fixed position. For the measurements in this paper, we used the second method (see the region of interest marked by the red lines in Figure 8). Next, all the scale marks are read along the selected line (see the green line in Figure 8) using the LabVIEWs built-in Advance Edge Tool edge detection procedure, which is based on finding the maxima and minima of a Fourier transform of the first derivative of grey level intensities along the given direction. Considering the finite thickness of each mark, their centreline is determined as the central position between the detected upper and lower bounds. Scale marks are indexed from the bottom up, starting at the first detected scale mark with index *i* = 0. The vertical distance of each scale mark from the centre of the image (*r_i_*) is measured in pixels. The uncertainty of the scale mark detection also depends on the vertical image resolution; the scale division equals approximately 5.75 pixels for the present system configuration.

Step 3: Liquid Level Detection

The liquid level is also determined by the above-mentioned LabVIEWs built-in edge detection procedure. The edge detection is carried out on multiple vertical lines across the fluid area (the blue lines in Figure 8), in order to eliminate the outliers that are caused by the effects of discrete air bubbles or droplets that might be present in the observed area. The meniscus is searched for in the bottom-up direction; in this way, the lowest point of the meniscus is detected (the purple line in Figure 8), and the pixel value of that position *r_liquid_* is determined.

The correct reading of the meniscus with the machine vision system was found to be more challenging when the liquid level was significantly below the camera centreline. In addition, the current tests were carried out with water, for which the detection of the liquid level is generally more difficult and more susceptible to the lighting conditions than in the case of coloured fluids (i.e., motor fuels). However, based on repeated tests also taking into account the camera being positioned at different vertical positions (heights), it was estimated that the uncertainty of the meniscus reading was better than a 0.75 scale interval (≈7.5 mL).

Step 4: Lens Correction

Lens distortion typically results in changes of the dimensional ratios of the image in the form of a barrel, pincushion distortions or combination of both [6]. Distortion is basically a two-dimensional effect and many algorithms for its correction have already been proposed, e.g., [18,19]. In our case, we describe the lens distortion as one-dimensional, because the lens correction parameters can be calculated using the positions of the scale marks (representing distortion-free values) detected along the given vertical line (see Figure 8). The dependency between the scale mark index (*i*) and the vertical pixel distance from the image centre (*r_i_*) was found to be well approximated by a cubic polynomial function:

(9) $$i\left(r\right)=Ar^{3}+Br^{2}+Cr+D.$$

**Figure 9:**
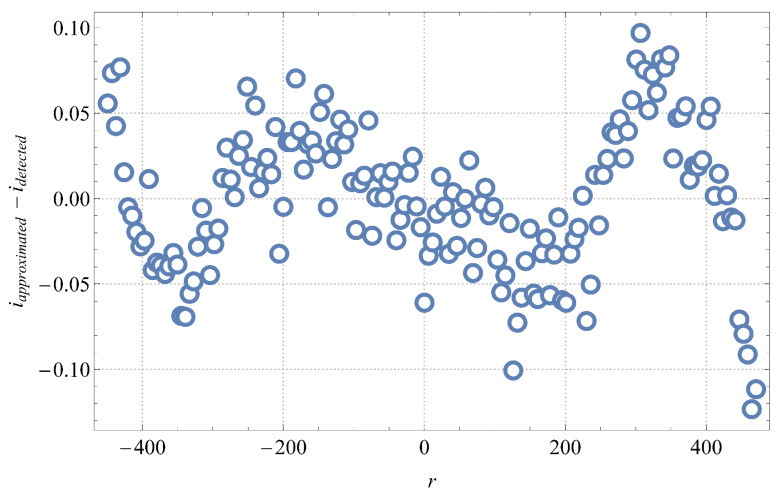
Deviation between the approximated and the detected scale mark indexes versus their pixel positions.

The difference between the approximated and the detected scale mark indexes versus their pixel positions is, for one case of our experiment, shown in Figure 9. It can be seen that the quality of the applied cubic approximation is approximately 0.1 of the scale interval (about 1 mL).

Finally, the liquid level *L*_1_ (as defined in Figure 1 or Figure 5) is calculated from the detected pixel value of the liquid level *r_liquid_* as follows:

(10) $$L_{1}=\left(i\left(r_{liquid}\right)-i\left(0\right)\right)k_{1}.$$

where *k*_1_ is the scale division in the length unit.

Step 5: Parallax Correction

Since the camera was positioned perpendicular to the measuring scale (γ = 0°), the parallax correction was based on Equation (4). The values of all parameters appearing in this equation are given in Table 1. The estimated relative uncertainty of the liquid level (*L*_3_) related to the measured distances, glass thickness, and its index of refraction is approximately 0.16%, i.e., approximately 0.13 mm (or approximately 1.3 mL) at the boundaries of the measuring range (−800 to 800 mL relative to the nominal capacity of 100 L) covered in the conducted experiment (see Section 4). The effect related to the camera tilt correction given in Equation (8) is, in our case, where tilt is believed to be smaller than 0.5°, estimated to be at most 0.05 mm across the measuring scale (see also Figure 5).

Step 6: Zeroing and Unit Conversion

Up to this point, we determined the liquid level *L*_3_ as a distance from the image centre. Finally, this result is converted to the (volume) reading of the capacity measure, i.e., the deviation from the nominal capacity ∆*V*. The computer program allows the user to preliminarily identify the scale mark which corresponds to the nominal volume or ∆*V* = 0 (index *i*_0_). Its distance from the image centre is calculated according to Equation (10), $L_{0}=\left(i_{0}-i\left(0\right)\right)k_{1}$. Then, the reading of the capacity measures is determined as:

(11) $$\Delta V=\left(L_{3}-L_{0}\right)k_{2}/k_{1}.$$

where *k*_2_ is the measuring scale division.

## 4. Results and Discussion

**Figure 10:**
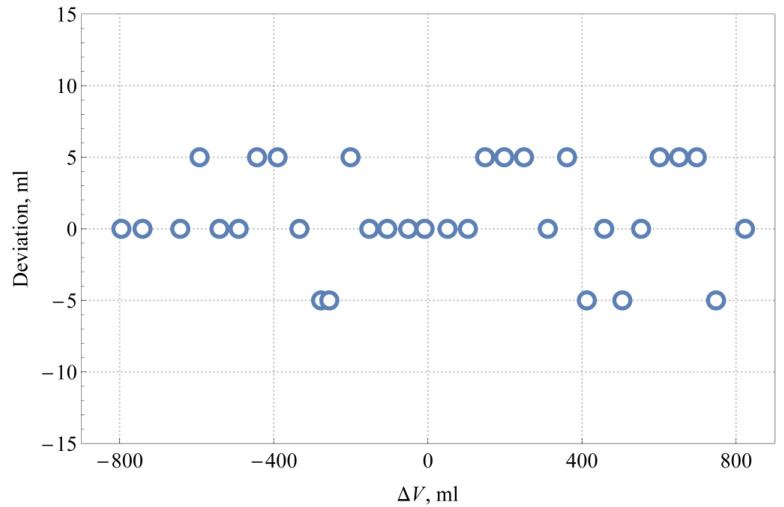
Deviation between the operators’ readings for different liquid levels.

The validation of the machine vision system was performed in a laboratory environment using the measuring system described earlier. The standard capacity measure was gradually filled with water in the range of scale marks from −800 to 800 mL in steps of approximately 50 mL, where the scale marks represent the deviation relative to its nominal capacity of 100 L. The readings of liquid levels were taken by two operators (operator 1 and 2), as well as by using the machine vision system. The maximum error of the reading of the liquid level by the operator is estimated to be at most half of the scale division (5 mL), which is also in accordance with the observed deviation of the operators’ readings shown in Figure 10.

The uncertainty of the machine vision measuring system can be estimated from relevant uncertainty components discussed in steps 2 to 5 of Section 3: (i) parallax correction, (ii) lens distortion, (iii) image resolution and (iv) the reading of the meniscus. The uncertainty of the meniscus reading is at least four times greater than all other contributions; therefore, the overall expanded uncertainty of the machine vision can be estimated to be equal to 7.5 mL (*k* = 2).

**Figure 11:**
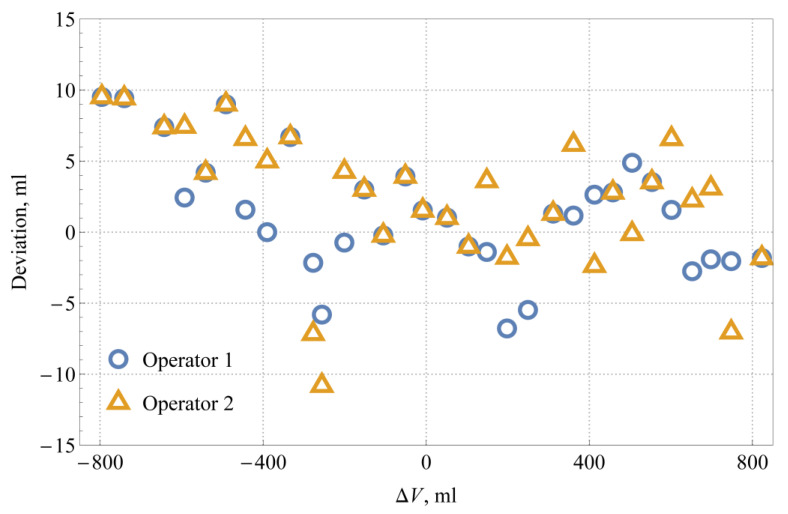
Deviations between the readings of the machine vision system and the operators for different liquid levels.

The deviation between the results read by the machine vision system and the operators for different liquid levels are shown in Figure 11. It is evident that only at one measuring point, the deviation between the measurement results of the automated system and the operators exceeded 10 mL. The shown deviations seem random, except maybe in the range ∆*V* < −400 mL, where the liquid levels detected by the automated system are always higher than those read by the operators, but do not exceed 10 mL.

The average value of the deviations shown in Figure 11 is 1.9 mL, and the experimental standard deviation is 4.5 mL. Considering the estimated maximum reading error by the operator of 5 mL (a half-width of the rectangular distribution) and the estimated expanded uncertainty of the automated system of 7.5 mL (a coverage factor *k* = 2), the standard uncertainty of the deviation can be—according to JCGM 100:2008 [20]—calculated as $\sqrt{{\left(5\;\mathrm{mL}/\sqrt{3}\right)}^{2}+{\left(7.5\;\mathrm{mL}/2\right)}^{2}}=4.7\;\mathrm{mL}$, which corresponds very well to the actual dispersion of the results. The results show the comparability of the automated system with the human operator and prove that the quality of readings with the machine vision system is certainly good enough for applications in the legal metrology, where the target expanded uncertainties are of the order of 0.1% (see discussion in Section 1), i.e., 100 mL for the 100 L capacity measure.

## 5. Conclusions

The present article focuses on the automation of the reading process of standard capacity measures, which represents commonly used volume standards for the testing and inspection of measuring systems for liquids other than water. We implemented a low-cost machine vision measuring system with a single fixed digital web camera that was positioned laterally to the transparent neck of the capacity measure. The measured quantity was determined by the digital processing algorithm in the LabVIEW programming environment, which includes the detection of the scale marks and the liquid meniscus with integrated edge detection algorithms, and corrections of the lens distortion and the parallax effects. The theory of the latter was discussed in detail. The testing and validation of the developed measuring system was performed by comparing its reading of the liquid level in a standard capacity measure filled with water with the readings made by the operators. The results showed no significant systematic errors, which confirms the proper setup of the measuring system and adequate treatment of its influential factors. The dispersion of the comparison results was of the same order as the estimated reading uncertainties. The developed measuring system reduces the time needed for measurement compared to the human operator and facilitates the reading of the liquid level in places that are difficult to access.

The tests confirmed the appropriateness of the presented machine vision measuring system for the field of legal metrology. However, to ensure the quality of the measured values, the tilt of the camera should be set to zero (within permissible deviation for the system under interest), and special care must be taken to provide suitable lighting conditions. These have a significant effect on the edge detection (of the meniscus), which proves important when dealing with transparent fluids and capacity measures with narrow necks, where the surface tension effect is greater, and the related rounding of the meniscus is more pronounced. Even if the measuring object is naturally lit, the use of an artificial light source is recommended, because it can ensure more constant lighting conditions and, therefore, a more stable output from the measuring system.

The presented machine vision system can also be used with liquids other than water. It was found that it is even less susceptible to lighting conditions if applied to measure the levels of nontransparent liquids, e.g., motor fuels, because a much clearer edge of the liquid can be obtained in such cases, even in suboptimal lighting conditions. Future improvements in the measuring system can be made by automating a detection of the camera tilt angle, which might be possible from its effect on the distortion of the scale mark readings.

## Figures and Tables

**Table 1:** Parameters of the experimental setup and their estimated uncertainties (*k* = 2).

*d*—distance between the camera and the measuring scale	(238 ± 2) mm
*d_s_*—distance between the measuring scale and the glass	(5.0 ± 0.2) mm
*d_g_*—thickness of the glass	(5.0 ± 0.5) mm
*n*_1_—refractive index of air	1
*n*_1_—refractive index of glass	1.52 ± 0.05
*k*_1_—distance between the scale marks	(1.0100 ± 0.0025) mm
*k*_2_—measuring scale division	10 mL
γ—tilt angle of the camera	0° ± 0.5°
